# Penile duplication (diphallia) with epispadias, neurogenic bladder, and lumbar vertebral Fusion: An exceptionally rare multisystem congenital association

**DOI:** 10.1016/j.eucr.2025.103169

**Published:** 2025-08-19

**Authors:** Narjes Saberi, Farid Rajaei Rizi, Mohammad Nozari

**Affiliations:** a Department of Urology, Isfahan Kidney Disease Research Center, Isfahan University of Medical Sciences, Isfahan, Iran; b Endocrine and Metabolism Research Center, Isfahan University of Medical Sciences, Isfahan, Iran; c Department of Urology, Al-Zahra Hospital, Isfahan University of Medical Sciences, Isfahan, Iran

**Keywords:** Penile duplication, Epispadias, Neurogenic bladder, Vertebral fusion, Case report

## Abstract

We report a highly unusual case of penile duplication (diphallia) accompanied by epispadias, neurogenic bladder with markedly reduced compliance, and lumbar vertebral fusion. Comprehensive clinical, imaging, and urodynamic examinations delineated complex genitourinary and spinal anomalies necessitating multidisciplinary management. The discussion reviews embryological implications, compares existing literature, and addresses long-term follow-up considerations. This case underscores the clinical and academic importance of individualized evaluation and tailored intervention for exceedingly rare multisystem congenital malformations.

## 1 Introduction

Penile duplication (diphallia) is an extremely rare congenital anomaly, estimated to occur in approximately 1 in 5.5 million live births, with few reported cases.^1^ The extent of duplication varies from isolated duplications to complex forms associated with other congenital anomalies, most frequently affecting the genitourinary, gastrointestinal, and musculoskeletal systems.^2^^,^^3^ Neurogenic bladder, often due to an underlying spinal dysraphism or vertebral anomaly, presents a significant risk for upper urinary tract deterioration, especially in pediatric populations.^4^^,^^5^ Early diagnosis and multidisciplinary management are crucial to preserving renal function and optimizing continence and quality of life in these complex cases.^6^ Herein, we report a unique case of complete penile duplication coexisting with epispadias, neurogenic bladder, and congenital lumbar vertebral fusion in an adolescent boy, and discuss the intricate diagnostic and therapeutic challenges encountered.

## 2 Case presentation

**Fig. 1:**
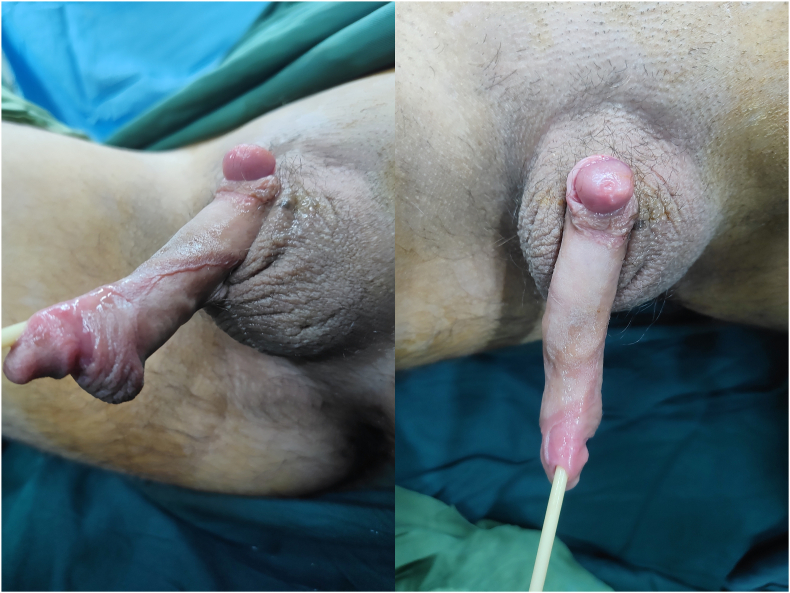
Comprehensive genital examination revealing complete penile duplication (true diphallia). The primary phallus demonstrates typical size with epispadias (dorsal urethral meatus) with successful urethral catheterization. The secondary phallus exhibits severe hypoplasia with complete urethral atresia despite systematic probing attempts. Symmetric scrotal development with bilateral descended testes.

A 14-year-old boy was referred to our pediatric urology clinic for lifelong continuous urinary incontinence and a congenital genital anomaly. The late referral was attributable to the patient's residence in a remote, underserved area with limited access to specialized healthcare and significant socioeconomic constraints. Detailed maternal history revealed full-term gestation with normal prenatal ultrasound findings. Developmental milestones were achieved normally until toilet training phase when persistent urinary incontinence prompted further investigation. The patient had persistent nocturnal enuresis with concomitant daytime urinary incontinence, without constipation or bowel evacuation dysfunction. Initial management included timed voiding, restriction of bladder irritants, and initiation of appropriate pharmacologic therapy. Notable absence of cutaneous spinal stigmata despite underlying vertebral fusion, and there was no family history of urological or neurological disease. On physical examination, he was well-nourished with normal vital signs. Genital inspection revealed complete penile duplication (diphallia). A phallus of normal size, with a dorsal meatus consistent with epispadias; a catheter passed easily into the bladder. Another phallus markedly hypoplastic and non-patent (Fig. 1). Both testes were descended in a normal scrotum. The perineum and anus were normal. Mild lumbar scoliosis was noted, with a firm prominence palpated at the L5 vertebral level. Neurological examination of the lower limbs—motor strength, sensation, and deep tendon reflexes—was intact.

**Fig. 2:**
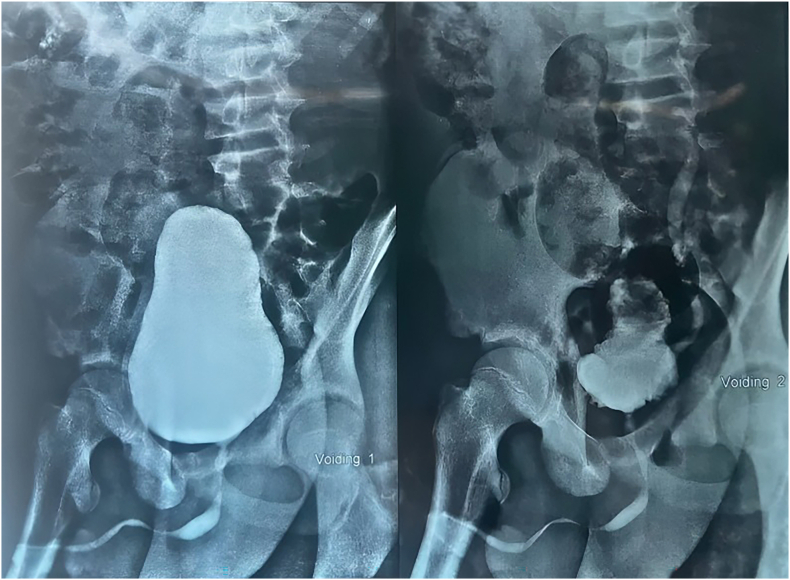
Following retrograde filling of the bladder with contrast medium, grade II vesicoureteral reflux was observed on the left side. The bladder wall appears smooth. The urethra is unremarkable except for mild epispadias. No residual urine was seen after voiding. A small suspected diverticulum is noted on the right side of the bladder.

Voiding cystourethrogram (VCUG) showed verticalization of bladder (elongated, more vertically oriented bladder with a blunted bladder neck–outlet angle, typically associated with neurogenic lower urinary tract dysfunction) and grade 2 vesicoureteral reflux in the left side (Fig. 2).

**Fig. 3:**
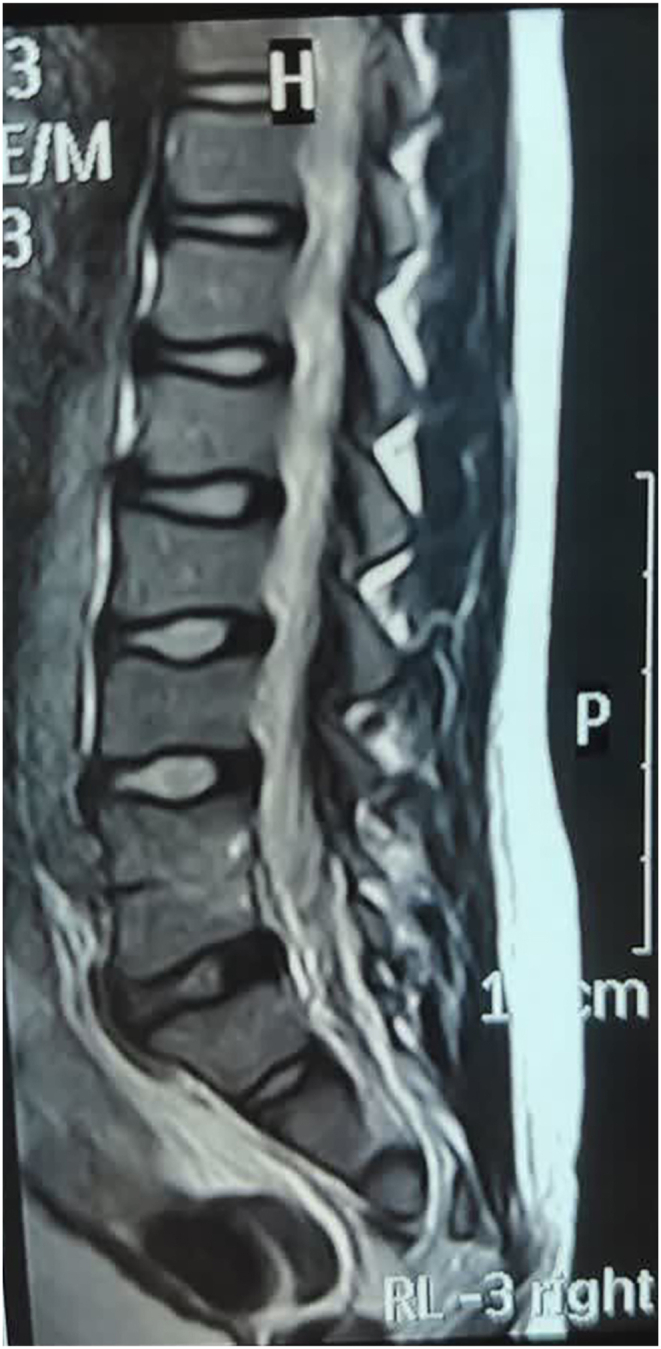
Sagittal and axial T2-weighted magnetic resonance imaging (MRI) demonstrates partial fusion of the L3 and L4 vertebral bodies (congenital block vertebra), with associated reduction in intervertebral disc height. The spinal cord terminates at a normal level without evidence of tethering or syringomyelia.

There were no clinical or urodynamic features suggestive of severe dysfunctional voiding or Hinman–Allen syndrome, and no evidence of associated bowel dysfunction. Lumbosacral MRI confirmed congenital fusion of the lumbar vertebral bodies. MRI demonstrated reduced intervertebral space at the lumbar level, which may indicate chronic compression of the spinal cord and serve as a plausible etiology for the neurogenic bladder. (Fig. 3).

**Fig. 4:**
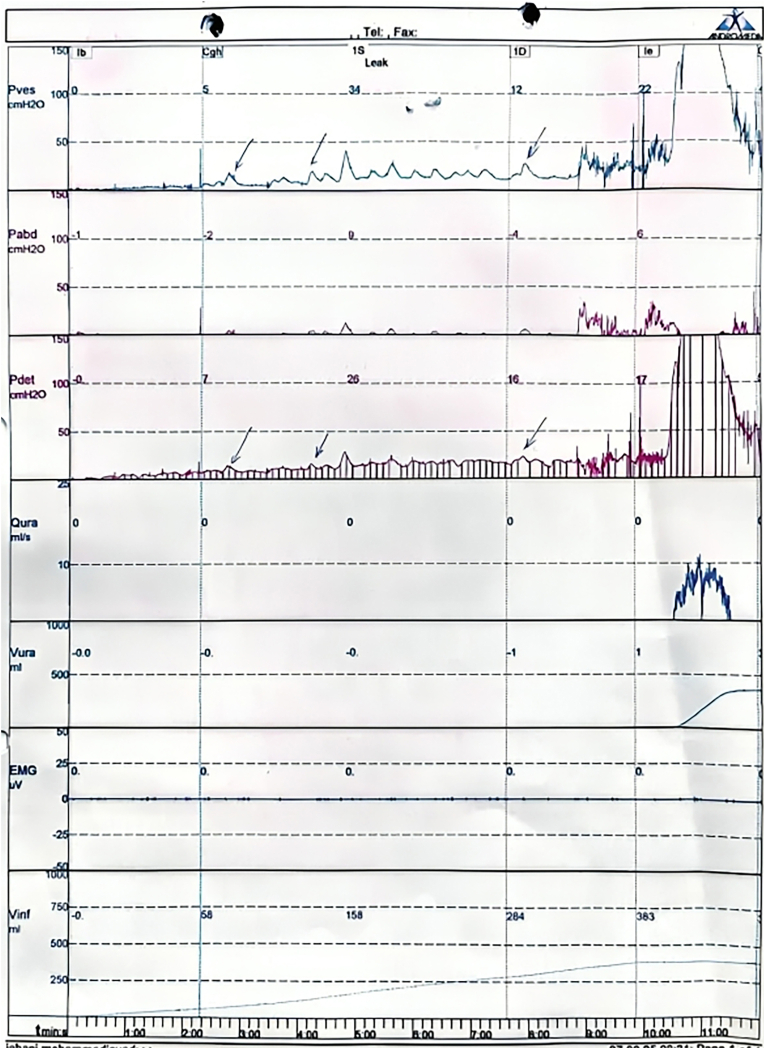
Representative urodynamic tracing showing normal bladder capacity with low compliance (<20 ml/cm H_2_O) and multiple (>10) phasic detrusor overactivity events during the filling phase, without leakage. Voiding phase illustrates high detrusor pressure (>100 cm H_2_O) and low urinary flow (<10 mL/sec), indicative of detrusor sphincter dyssynergia. The EMG channel was disconnected due to a technical error, and the patient declined repeat testing due to discomfort.

The patient's initial management, instituted at diagnosis, included clean intermittent catheterization (CIC) four times daily to maintain low intravesical pressures, alongside oral oxybutynin at a dose of 0.3 mg/kg/day (administered in two divided doses) to control detrusor overactivity. Only one urodynamic study was performed, three months after initiation of clean intermittent catheterization (CIC) and conservative management. Despite strict adherence to this regimen, urodynamic study at three months revealed persistently elevated bladder storage pressures and ongoing detrusor overactivity, with continued daily incontinence. Urodynamic evaluation demonstrated a normal bladder capacity but abnormally low compliance (<20 ml/cm H_2_O) during the filling phase, accompanied by multiple (>10) episodes of phasic detrusor overactivity without involuntary leakage. During the voiding phase, the study revealed a high-pressure pattern (maximum detrusor pressure >100 cm H_2_O) with low urinary flow rates (<10 mL/sec), strongly suggestive of detrusor sphincter dyssynergia. These findings collectively indicated a high-risk neurogenic bladder with both storage and voiding dysfunction, underscoring the necessity for aggressive and multidisciplinary management (Fig. 4).

**Fig. 5:**
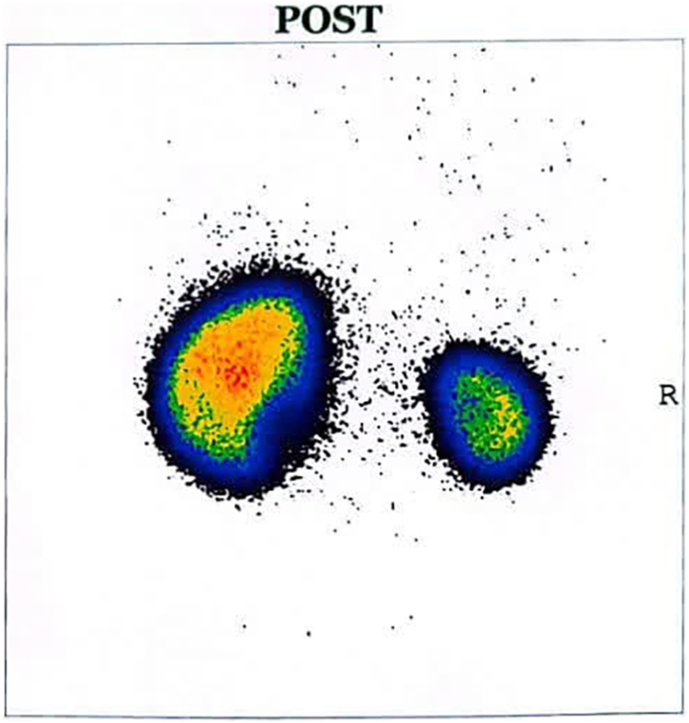
99mTc-DMSA renal scan showing severe global cortical loss and mild border irregularity in the small right kidney, with markedly decreased function. The left kidney displays decreased uptake in the lower pole and lower medial segment, with mild border irregularity. Differential function: right kidney 24.66 %, left kidney 74.35 %.

A 99mTc-DMSA renal scan was performed to further evaluate cortical integrity and differential function. The DMSA findings revealed severe global cortical loss in the small right kidney, which also exhibited mildly irregular borders. The left kidney demonstrated decreased cortical uptake in the lower pole and lower medial segment, along with mild border irregularity. Differential renal function was significantly impaired, with the right kidney contributing only 24.66 % and the left kidney 74.35 % of total renal function (Fig. 5).

In view of refractory high-pressure neurogenic bladder—posing a clear risk to upper urinary tract integrity—a second-line intervention was undertaken at the three-month mark. The patient underwent intradetrusor injection of 300 units of onabotulinumtoxinA, distributed across 20–25 sites under general anesthesia. The procedure was well tolerated, and no acute complications were observed.

Karyotype analysis revealed a normal 46, XY chromosomal pattern in our patient, which is consistent with the findings in most diphallia case reports.

## 3 Follow-up and outcomes

4 Weeks Post-Injection: Patient reported 1–2 leakage episodes per day (down from continuous leakage) and subjective improvement in bladder comfort, the patient's follow-up care was incomplete due to inadequate adherence to scheduled evaluations. Regarding the penile duplication, the patient was referred to a reconstructive urologist for further evaluation. Surgical intervention has been deferred at this stage, with priority given to preserving renal function; the ultimate decision will be made jointly by the surgical team, patient, and family.

## 4 Discussion

Penile duplication (diphallia) is an extraordinarily rare congenital malformation, estimated to occur in approximately 1 in 5.5 million live births.^7^^,^^8^ The clinical spectrum is broad, ranging from partial duplication (bifid phallus) to true complete diphallia associated with varying degrees of genital, urinary, and anorectal structural anomalies. The condition is often accompanied by various urogenital, gastrointestinal, and musculoskeletal anomalies, demanding individualized management strategies based on the anatomical and functional specifics of each case.^9^

The embryology of diphallia remains incompletely understood, but current theories implicate duplication or incomplete fusion of the genital tubercle and cloacal membrane during early mesodermal development.^10^ The coexistence of true complete penile duplication, epispadias, neurogenic bladder, and vertebral fusion is so rare that only isolated, single-case mentions exist in the literature. Review of published data suggests these anomalies may stem from early errors in mesodermal development and cloacal membrane partitioning—potentially from disruption during the formation of the genital tubercle and notochord.^10^

Our patient presented with complete penile duplication, epispadias, and a hypoplastic phallus, alongside descended testes (Fig. 1). Initial imaging, including voiding cystourethrogram (VCUG), revealed bladder verticalization (more upright orientation of the bladder outlet axis) and grade 2 vesicoureteral reflux. Untreated neurogenic bladder in children, especially in the context of spinal dysraphism, carries substantial risks of upper urinary tract deterioration and incontinence.

Consistent with current guidelines, initial management involved clean intermittent catheterization (CIC) and anticholinergic therapy, aimed at protecting renal function and achieving safe bladder storage.^11^ Despite these interventions, follow-up urodynamic studies (UDS) demonstrated persistent detrusor overactivity and elevated storage pressures (Fig. 4), predicting unfavorable outcomes for both continence and renal preservation. A subsequent DMSA scan further delineated severe global cortical loss in the right kidney and diminished function in the contralateral kidney (Fig. 5), underscoring the critical need for more effective intervention. Consequently, intravesical onabotulinumtoxinA (Botox) injection was employed as a second-line intervention.

Given the complex interplay of anatomical and functional challenges, multidisciplinary management is mandatory. While CIC remains first-line in the absence of severe anatomic obstruction or intractable incontinence, alternative surgical interventions must be planned proactively. Options include.

• **Augmentation cystoplasty**: Indicated in cases of refractory poor compliance or persistent upper tract changes, providing increased bladder capacity and reducing pressures.
• **Mitrofanoff appendicovesicostomy**: Offers a continent catheterizable channel, especially when urethral access becomes impractical or social continence is desired.
• **Urinary diversion**: Reserved for failed reconstructive efforts or when bladder preservation is not feasible.^8^

This case brings several key insights:

**Diagnostic implication**: Such rare constellations necessitate high clinical vigilance, exhaustive systemic workup, and cross-disciplinary collaboration, ensuring that no component anomaly is overlooked.

**Scientific/embryological impact**: Each report incrementally strengthens the global knowledge base, enabling refined genetic mapping and mechanistic models for organogenesis failures.

**Practice message**: Multisystem malformation in diphallia maybe should prompt mandatory spine and renal assessment. This case underscores the importance of measures to ensure long-term follow-up of children at risk and the need to educate primary care providers in recognizing genital anomalies promptly to prevent delayed diagnosis and intervention.

## 5 Conclusion

This report highlights the critical importance of comprehensive anatomical and functional evaluation in rare genitourinary anomalies. The unique constellation of diphallia, epispadias, neurogenic bladder, and vertebral fusion underscores embryological interrelationships and the necessity for individualized, multidisciplinary care. In summary, our case expands the phenotypic spectrum of diphallia and exemplifies the critical need for high-index clinical suspicion, systematic embryological and functional assessment, and tailored multidisciplinary care. Further multicenter studies and genetic investigations are warranted to elucidate underlying mechanisms and optimize long-term management of these exceptionally rare multisystem congenital anomalies.

## 6 Limitations

This report is subject to several limitations. First, the duration of follow-up after intravesical onabotulinumtoxinA (Botox) injection is relatively short, precluding definitive assessment of the long-term durability of the therapeutic effect. Second, advanced genetic analyses beyond standard karyotyping were not performed, so underlying syndromic or molecular etiologies cannot be excluded. Third, as with all single-case reports, the findings cannot be generalized to broader patient populations or used to inform definitive etiological or therapeutic strategies. Fourth, there is insufficient data thus far regarding the potential future need for surgical interventions such as bladder augmentation or creation of a continent catheterizable channel (e.g., Mitrofanoff procedure).

Finally, the patient's follow-up care was incomplete due to inadequate adherence to scheduled evaluations, limiting our ability to fully assess long-term outcomes and the evolving effectiveness of conservative management. Ongoing surveillance will be necessary to better evaluate the durability of conservative or minimally invasive management and to inform long-term outcomes.

## CRediT authorship contribution statement

**Narjes Saberi:** Writing – review & editing, Writing – original draft, Supervision. **Farid Rajaei Rizi:** Writing – review & editing, Writing – original draft, Visualization, Validation, Supervision, Software, Resources, Project administration, Methodology, Investigation, Funding acquisition, Formal analysis, Data curation, Conceptualization. **Mohammad Nozari:** Writing – review & editing, Writing – original draft.

## Ethics approval and consent to participate

Written informed consent was obtained from the patient's parent for publication of this case report and accompanying images.

## Use of artificial intelligence

Artificial intelligence (AI) was used solely for text refinement and English language editing during manuscript preparation. All scientific content, analysis, and clinical interpretation were provided by the authors.

## Competing interests

The authors declare no conflicts of interest related to this manuscript.

## References

[1] Diphallia with associated anomalies: a case report and literature review - PMC. https://pmc.ncbi.nlm.nih.gov/articles/PMC3870645/.

[2] Mirshemirani A.R., Sadeghyian N., Mohajerzadeh L., Molayee H., Ghaffari P. (2010). Diphallus: report on Six cases and review of the literature. Iran J Pediatr.

[3] Al-Abbasi B.K. (2022). Complete diphallia associated with unusual multiple congenital anomalies: case report and review of literatures. Annals of Pediatric Surgery.

[4] Hobbs K.T., Krischak M., Tejwani R., Purves J.T., Wiener J.S., Routh J.C. (2021). The importance of early diagnosis and management of pediatric neurogenic bladder dysfunction. Res Rep Urol.

[5] Sturm R.M., Cheng E.Y. (2016). The management of the pediatric neurogenic bladder. Current Bladder Dysfunction Reports.

[6] Evaluation and long-term management of neurogenic bladder in spinal dysraphism | NeoReviews | American Academy of Pediatrics. Accessed July 24, 2025. https://publications.aap.org/neoreviews/article-abstract/20/12/e711/92013/Evaluation-and-Long-term-Management-of-Neurogenic?redirectedFrom=fulltext. 10.1542/neo.20-12-e711 31792158

[7] Kendrick D.J., Kimble R.M. (2022). Diphallia: literature review and proposed surgical classification system. ANZ J Surg.

[8] Gyftopoulos K., Wolffenbuttel K.P., Nijman R.J.M. (2002). Clinical and embryologic aspects of penile duplication and associated anomalies. Urology.

[9] Vafaei H., Roozmeh S., Bahador A., Khafri M.Z., Ghiasi M. (2022). Prenatal diagnosis of diphallia in association with bladder exstrophy: a case report. BMC Pregnancy Childbirth.

[10] (2023). A case report on penile duplication: a rare congenital anomaly - Rao Nouman Ali, Sohaib Irfan, Wajiha Irfan. https://journals.sagepub.com/doi/full/10.1177/20514158211066419?casa_token=DSkhh-Ms9AEAAAAA%3A_pb3wLx3_HIvClGQEFtwz1LH0u0YSuQouhxvXPWia_UCFz6qw3Zg6VJXh5gwoOJXJT0xzw4C0wvnwg.

[11] Urological science. https://journals.lww.com/ursc/fulltext/2023/34010/clinical_guidelines_of_patient_centered_bladder.3.aspx.

